# Plasma and cerebrospinal fluid cholesterol esterification is hampered in Alzheimer’s disease

**DOI:** 10.1186/s13195-023-01241-6

**Published:** 2023-05-20

**Authors:** Marta Turri, Elisa Conti, Chiara Pavanello, Francesco Gastoldi, Marcella Palumbo, Franco Bernini, Vittoria Aprea, Francesca Re, Alberto Barbiroli, Davide Emide, Daniela Galimberti, Lucio Tremolizzo, Francesca Zimetti, Laura Calabresi, AGAINST-AD Group, Simona Andreoni, Ildebrando Appollonio, Fulvio Da Re, Carlo Ferrarese, Aristotelis Karantzoulis, Giulia Negro, Federico Emanuele Pozzi, Giulia Remoli, Benedetta Storti, Chiara Paola Zoia

**Affiliations:** 1 Centro E. Grossi Paoletti, Dipartimento Di Scienze Farmacologiche E Biomolecolari, Università Degli Studi Di Milano, Milano, Italy; 2 Neurology Unit, IRCCS “San Gerardo Dei Tintori”, Monza, and University of Milano-Bicocca, Milano, Italy; 3 Department of Food and Drug, University of Parma, Parma, Italy; 4 School of Medicine and Surgery, University of Milano-Bicocca, Milano, Italy; 5 Dipartimento Di Scienze Per Gli Alimenti, La Nutrizione E L’Ambiente, Università Degli Studi Di Milano, Milano, Italy; 6 Department of Biomedical, Surgical and Dental Sciences, University of Milan, Milan, Italy; 7 Fondazione IRCCS Ca’ Granda, Ospedale Policlinico, Milan, Italy

**Keywords:** Alzheimer’s disease, High-density lipoproteins, Lecithin:cholesterol acyltransferase, Cholesterol efflux, Apolipoprotein E

## Abstract

**Objective:**

The purpose of this study was to evaluate cholesterol esterification and HDL subclasses in plasma and cerebrospinal fluid (CSF) of Alzheimer’s disease (AD) patients.

**Methods:**

The study enrolled 70 AD patients and 74 cognitively normal controls comparable for age and sex. Lipoprotein profile, cholesterol esterification, and cholesterol efflux capacity (CEC) were evaluated in plasma and CSF.

**Results:**

AD patients have normal plasma lipids but significantly reduced unesterified cholesterol and unesterified/total cholesterol ratio. Lecithin:cholesterol acyltransferase (LCAT) activity and cholesterol esterification rate (CER), two measures of the efficiency of the esterification process, were reduced by 29% and 16%, respectively, in the plasma of AD patients. Plasma HDL subclass distribution in AD patients was comparable to that of controls but the content of small discoidal preβ-HDL particles was significantly reduced. In agreement with the reduced preβ-HDL particles, cholesterol efflux capacity mediated by the transporters ABCA1 and ABCG1 was reduced in AD patients’ plasma. The CSF unesterified to total cholesterol ratio was increased in AD patients, and CSF CER and CEC from astrocytes were significantly reduced in AD patients. In the AD group, a significant positive correlation was observed between plasma unesterified cholesterol and unesterified/total cholesterol ratio with Aβ_1-42_ CSF content.

**Conclusion:**

Taken together our data indicate that cholesterol esterification is hampered in plasma and CSF of AD patients and that plasma cholesterol esterification biomarkers (unesterified cholesterol and unesterified/total cholesterol ratio) are significantly associated to disease biomarkers (i.e., CSF Aβ_1-42_).

**Supplementary Information:**

The online version contains supplementary material available at 10.1186/s13195-023-01241-6.

## Background

Brain cholesterol metabolism is segregated from the peripheral circulation by the blood–brain barrier (BBB) and cholesterol transport in the central nervous system (CNS) is operated by lipoproteins very similar to the circulating high-density lipoproteins (HDLs) and called “HDL-like particles” [1]. Plasma HDLs have been extensively studied and characterized and consist of heterogeneous particles differing in shape, size, and composition [2]. The heterogeneity of circulating HDLs is the result of HDL remodeling by various enzymes and lipid transfer proteins. Among these factors, lecithin:cholesterol acyltransferase (LCAT), the enzyme responsible of cholesterol esterification in plasma, plays a major role in HDL maturation [3].

Brain HDLs are similarly heterogeneous in size and composition [1]; while in plasma the major HDL protein constituent is apolipoprotein A-I (apoA-I), the major protein component of brain HDL is apoE [1], which is synthesized in situ and does not derive from the circulation [1]. In humans, apoE exists as three different isoforms, apoE2, apoE3, and apoE4, which differ by amino acid substitutions at positions 112 and 158 in the mature protein [4]. ApoE3 contains cysteine at position 112 and arginine at position 158, whereas apoE2 and apoE4 contain cysteine or arginine, respectively, at both positions. ApoE3 is the most common isoform, whereas the apoE2 and apoE4 isoforms are less frequent and have altered functionality. ApoE4 has reduced lipoprotein binding and it has been associated with hypercholesterolemia and Alzheimer’s disease (AD) [1, 5–9]. Brain HDLs also contain apoA-I, which is not produced in the CNS but which, in the poorly lipidated form, can cross the BBB, likely through different mechanisms [10]. ApoJ, known as clusterin, is also synthesized in the brain and it is the second major apolipoprotein constituent of brain lipoproteins [11]. Brain HDL remodeling likely occurs by the action of the same enzymes and proteins operating in the circulation. LCAT, the major player in HDL maturation, is mainly synthesized by the liver but also in the brain, suggesting a crucial role of this enzyme in the maturation of brain lipoproteins, although LCAT concentration in the cerebrospinal fluid (CSF) is much lower than in plasma [12].

An inverse association between plasma HDL-cholesterol levels and the risk of dementia and AD has been reported, although with conflicting results [1], but an involvement of circulating HDLs in AD etiology and progression has never been proven. Few studies have analyzed HDL in the CSF, for obvious reasons of sample accessibility. Published data have mainly focused on the cholesterol efflux capacity (CEC) of CSF HDL and showed that it is reduced in AD patients [13, 14]; a very recent report has suggested that the CSF levels of apoJ are a determinant of the low CSF CEC observed in dementia [15].

In the present report, we have hypothesized that cholesterol esterification is altered in AD plasma and CSF. Due to the much lower concentration of lipids and enzymes in the CSF compared to plasma, we have set appropriate techniques to measure lipids, lipoproteins, apolipoproteins, and cholesterol esterification in the CSF.

## Material and methods

### Subjects

Seventy AD outpatients were recruited at the IRCCS “San Gerardo dei Tintori”, Monza (Italy). All patients were seen for the first time for clinical manifestations of cognitive symptoms and were evaluated to confirm or not AD diagnosis. AD was initially diagnosed according to the NINCDS-ADRDA criteria [16] and alternative diagnoses were excluded by brain MRI scan, a routine extensive neuropsychological test battery, and evaluation of CSF biomarkers. Only patients displaying a CSF A + T + N + profile [17] were recruited. Patients were recruited also only if the main caregiver was available for the interview. For each patient, the current score at the Mini-Mental State Examination (MMSE) and Clinical Dementia Rating (CDR) was obtained.

Seventy-four age- and sex-comparable cognitively normal controls (CN) without a personal or family history of neurological or psychiatric disorders were recruited at the IRCCS “San Gerardo dei Tintori,” Monza, the Center E. Grossi Paoletti and the Ospedale Policlinico in Milano (Italy). Lack of cognitive impairment in controls was established by a clinical interview, including a MMSE score > 26, CDR = 0, and by CSF biomarker profile in the sub-group of controls (*n* = 21) that underwent to CSF withdrawal for medical procedures not related to cognitive decline.

For all patients, pharmacological therapy was recorded, with specific attention to lipid-modifying drugs. Patients and controls with recent infections or surgery (6 months), or under anti-inflammatory, corticosteroid, or immunosuppressive drug treatments, or affected by kidney or liver failure were excluded.

The study was conducted in accordance with the guidelines of the Declaration of Helsinki and its later amendments and was approved by the internal ethical committee (Comitato Etico Brianza, approval #3267 of May 21, 2020) and all subjects signed an informed consent.

### Plasma and CSF sample preparation and *APOE* genotyping

Blood samples (15 mL) were collected from all patients and controls after over-night fasting and immediately centrifuged. Plasma aliquots were frozen at -80° C until blind assessments.

To analyze *APOE* genotype, total DNA was extracted from peripheral blood using a commercial DNA extraction kit (Qiagen, Venlo, Netherlands), and DNA amplification was performed using specific primers as previously reported [18].

CSF was obtained from all patients and from a sub-group of controls (*n* = 21) for whom CSF collection was indicated for medical procedures not related to cognitive decline. CSF was collected by lumbar puncture using a 21-gauge needle in 10-mL polypropylene tubes. Part of the CSF was used for routine analysis including leukocyte count, erythrocyte count, glucose concentration, and total protein concentration. Within 2 h, the remaining CSF was centrifuged at 2000 g for 10 min at room temperature to eliminate cells, transferred to new polypropylene tubes, and stored at − 80 °C until biomarker analysis.

Aβ_1-40_, Aβ_1-42_, T-tau, and P-tau were evaluated using commercially available kits Fujirebio© using the Lumipulse G600II instrument [19]. Cut-off values for AD diagnosis were the following (normal values are reported): Aβ_1-42_ > 599 pg/mL; Aβ_1-40_ n.a.; T-tau < 404 pg/mL; P-tau < 56.5 pg/mL; Aβ_1-42_/_1-40_ ratio > 0.069; Aβ_1-42_/T-tau ratio > 1.275; Aβ_1-42_/P-tau ratio > 8.1.

### Plasma biochemical analyses

A complete lipid-lipoprotein profile, including total cholesterol, HDL-cholesterol, triglycerides, apoA-I, apoA-II, apoE, and apoB, was determined using a Roche Integra c311 autoanalyzer. LDL-cholesterol was calculated by Friedewald’s formula. Plasma unesterified cholesterol and phospholipids were determined by enzymatic techniques. The amount of cholesteryl esters (CEs) was calculated by subtracting unesterified cholesterol from total cholesterol and the difference was multiplied by 1.68 to have a precise estimation of the CE mass. The cholesterol esterification process was evaluated in plasma samples by measuring the cholesterol esterification rate (CER), which reflects the ability of endogenous LCAT to esterify cholesterol within endogenous lipoproteins, and LCAT activity, which reflects the ability of endogenous LCAT to esterify cholesterol within exogenous reconstituted HDL [20].

### CSF biochemical analyses

Frozen CSF samples were slowly defrosted in ice at the moment of utilization, avoiding repeated freeze-thawing. To exclude blood contamination, all CSF samples were tested for apoB, which is not normally found in CNS [15]. CSF total and unesterified cholesterol were measured by HPLC. To quantify total cholesterol, an aliquot of CSF was incubated with cholesterol esterase (Sigma-Aldrich) for 1 h at 37 °C to convert all cholesterol into the unesterified form. Esterase-treated and esterase-untreated CSF aliquots were extracted in duplicate with 2-propanol (1:5, v:v). The extracts were then analyzed by RP-HPLC in isocratic conditions with a Jupiter 4u Proteo 90A 150 × 4.6 mm column. The mobile phase was acetonitrile:2-propanol (2:1, v:v) operating at 0.8 mL/min. The chromatograms were recorded at λ = 210 nm and cholesterol content was measured through a calibration curve (5–250 ng), constructed using standard cholesterol solubilized in 2-propanol. Total cholesterol was quantified from the esterase-treated aliquots, whereas unesterified cholesterol from the esterase-untreated aliquots.

Cholesterol esterification in the CSF was evaluated as plasma CER [20] with few adaptations due to the very low CSF concentration of substrate and enzyme. Specifically, CSF samples were incubated for 6 h instead of 1 h as in plasma, and unesterified cholesterol concentration was measured before and after incubation by HPLC as described above. The difference in unesterified cholesterol allowed the calculation of the CSF CER as nmol CE/mL/h.

ApoE and apoA-I were determined in CSF by SDS electrophoresis followed by immunodetection with specific antibodies (Calbiochem); increasing amounts of purified proteins were loaded and used to calculate standard curves. Phospholipids were measured with a commercial kit (Sigma-Aldrich).

### HDL subclass distribution

HDL subclasses were evaluated in plasma and CSF samples by two-dimensional electrophoresis, where agarose gel electrophoresis was followed by nondenaturing gradient gel electrophoresis and then immunoblotting to detect apoE (Calbiochem) and apoA-I (Calbiochem). Briefly, in the first dimension, 10 μL of plasma or CSF (concentrated 10 folds using Microcon ultra (Merk)) were separated by charge on a 0.5% agarose gel (Hydragel protein(e) kit, Sebia PN4120); agarose gel strips containing the pre-separated lipoproteins were then positioned onto a home-made 3–20% nondenaturing polyacrylamide gradient gel. Separated particles were then transferred onto a nitrocellulose membrane on which apoE-containing and apoA-I-containing lipoproteins were detected with anti-apoE and anti-apoA-I antibodies. The relative content of preβ-HDL was calculated by using the Bio Rad Multi-Analyst /PC Software and expressed as a percentage of total apoA-I.

### Plasma HDL cholesterol efflux capacity

Plasma HDL-cholesterol efflux capacity (CEC) was evaluated after isolating the HDL fraction of each plasma sample by precipitation of the apoB-containing lipoproteins with polyethylene glycol, as previously described [21, 22]. To avoid any lipoprotein remodeling, sera were slowly defrosted at 4 °C before the procedure.

The aqueous diffusion and the ABCA1-mediated CEC were evaluated in the murine macrophage cell line J774A.1 (Sigma-Aldrich, Milano, Italy) as previously described [23]. The aqueous diffusion CEC was evaluated in J774A.1 in basal conditions, while the ABCA1-mediated CEC was calculated as the difference between CEC from cells treated with cpt-cAMP (Merk Life Science, Milano, Italy) to induce ABCA1 expression [24] and the aqueous diffusion CEC.

The ABCG1-mediated HDL-CEC was evaluated in Chinese hamster ovary (CHO) cells transfected and not transfected with the human ABCG1 gene as previously described [23]. CHO-K1 cells (ATCC, Manassas, VA, USA) were used for stable transfection of hABCG1 [25]. The specific ABCG1 contribution was calculated as the difference between HDL-CEC in ABCG1-transfected and non-transfected cells.

### CSF cholesterol efflux capacity

For the evaluation of CSF-CEC, whole CSF was used since HDL-like particles have been identified as the sole lipoproteins present in this biological fluid [13, 14, 26]. To avoid any potential lipoprotein remodeling, CSF samples were slowly defrosted at 4 °C before use.

CSF-CEC was measured in human glioblastoma astrocytoma U373-MG cell line, a standard surrogate model for human astrocytes, with a radio-isotopic assay as previously described [27]. The cells were kindly donated by Prof. Ovidio Bussolati from the Department of Medicine and Surgery of the University of Parma (Italy) and authenticated through the Cell Line Authentication (CLA) service (Eurofins Genomic, Ebersberg, Germany). After seeding, cells were radiolabeled for 24 h with 2 µCi/mL of [1,2-^3^H(N)]-cholesterol (PerkinElmer, Waltham, MA, USA) in the presence of 2 µg/mL of an Acyl CoA: Cholesterol O-acyl transferase (ACAT) inhibitor compound (Sandoz; Sigma-Aldrich, Milano, Italy), to ensure all cholesterol present in the unesterified form. Subsequently, cells were treated for 18 h with DMEM supplemented with 0.2% bovine serum albumin (Sigma-Aldrich, Milano, Italy), the ACAT inhibitor, and the LXR/RXR agonists 22-hydroxycholesterol and 9-cis retinoic acid (5 µg/mL and 10 µM, respectively) to induce the expression of the active transporters ABCA1 and ABCG1 [27]. Finally, cells were exposed to CSF (30% v:v in DMEM) from AD patients and control subjects for 6 h. Cholesterol efflux was calculated as the percentage of radioactivity released into the medium over the total radioactivity incorporated by the cells.

### Statistical analysis

Numerical variables were summarized as mean ± SD, if normally distributed, or as median (1^st^ and 3^rd^ quartile) otherwise and were compared between groups by the Wilcoxon rank-sum test. Categorical variables were summarized as number (%) and were compared between groups by chi-square test or Fisher’s exact test, as appropriate. Associations between variables were assessed by Spearman’s rank correlation. Due to the large number of tests performed, *p*-values < 0.05 were considered as suggestive of an association, and *p*-values < 0.005 were considered as fully significant. All tests were two-sided and analyses were performed using SAS v. 9.4 (SAS Inc, Cary, NC, USA).

## Results

### Subject characteristics

The characteristics of the enrolled AD patients and cognitively normal controls are reported in Table 1. The two groups were comparable for age and sex distribution. AD patients had an average MMSE of 20.9 and CDR of 1.4, in line with initial clinical manifestations. Liquor markers were per protocol confirmatory of AD in all the enrolled patients. *APOE* genotype distribution in the AD group was different from that of the control group and characterized by a predominance of carriers of the *APOE* ε4 allele, as described in previous Italian AD cohorts [28, 29]. More than one fourth of AD patients was taking statins, while only 6.8% of controls was treated.

**Table 1:** Demographic and clinical data of Alzheimer’s disease patients and control subjects

	AD	CN
N	70	74
Sex (M/F)	34/36	37/37
Age (y)	73.6 ± 6.6	70.8 ± 9.8
MMSE (score)	20.9 ± 4.2	
CDR (score)	1.4 ± 0.7	
Disease biomarkers^a^		
Aβ_1-42_ (pg/mL)	442 (303; 572)	1,034 (608; 1,765)
Aβ_1-40_ (pg/mL)	10,482 (7838; 12,869)	n.a
T-tau (pg/mL)	673 (464; 965)	176 (93; 325)
P-tau (pg/mL)	100 (78; 147)	33 (24; 48)
Aβ_1-42_/Aβ_1-40_	0.044 (0.038; 0.051)	n.a
Aβ_1-42_/T-tau	0.67 (0.36; 1.09)	6.40 (2.01; 14.53)
Aβ_1-42_/P-tau	4.4 (2.5; 6.3)	30.34 (13.78; 53.13)
Apolipoprotein E genotype (*N*, %)^b^		
E4/E4	4 (5.8)	2 (5.9)
E3/E4	30 (43.5)	4 (11.8)
E3/E3	33 (47.8)	25 (73.5)
E2/E3	2 (2.9)	3 (8.8)
Statins (*N*, %)	19 (27.1)	5 (6.8)

*AD* Alzheimer’s disease, *CN* cognitive normal controls, *MMSE* Mini-Mental State Examination, *CDR* Clinical Dementia Rating Scale

Data are reported as mean ± SD or median (1^st^ quartile; 3^rd^ quartile), as appropriate, or *N* (%) for categorical variables

^a^Disease biomarkers were available for 21 controls

^b^Apolipoprotein E genotype was evaluated in 69 AD patients and in 34 controls

### Plasma lipid/lipoprotein profile in AD patients

**Fig. 1:**
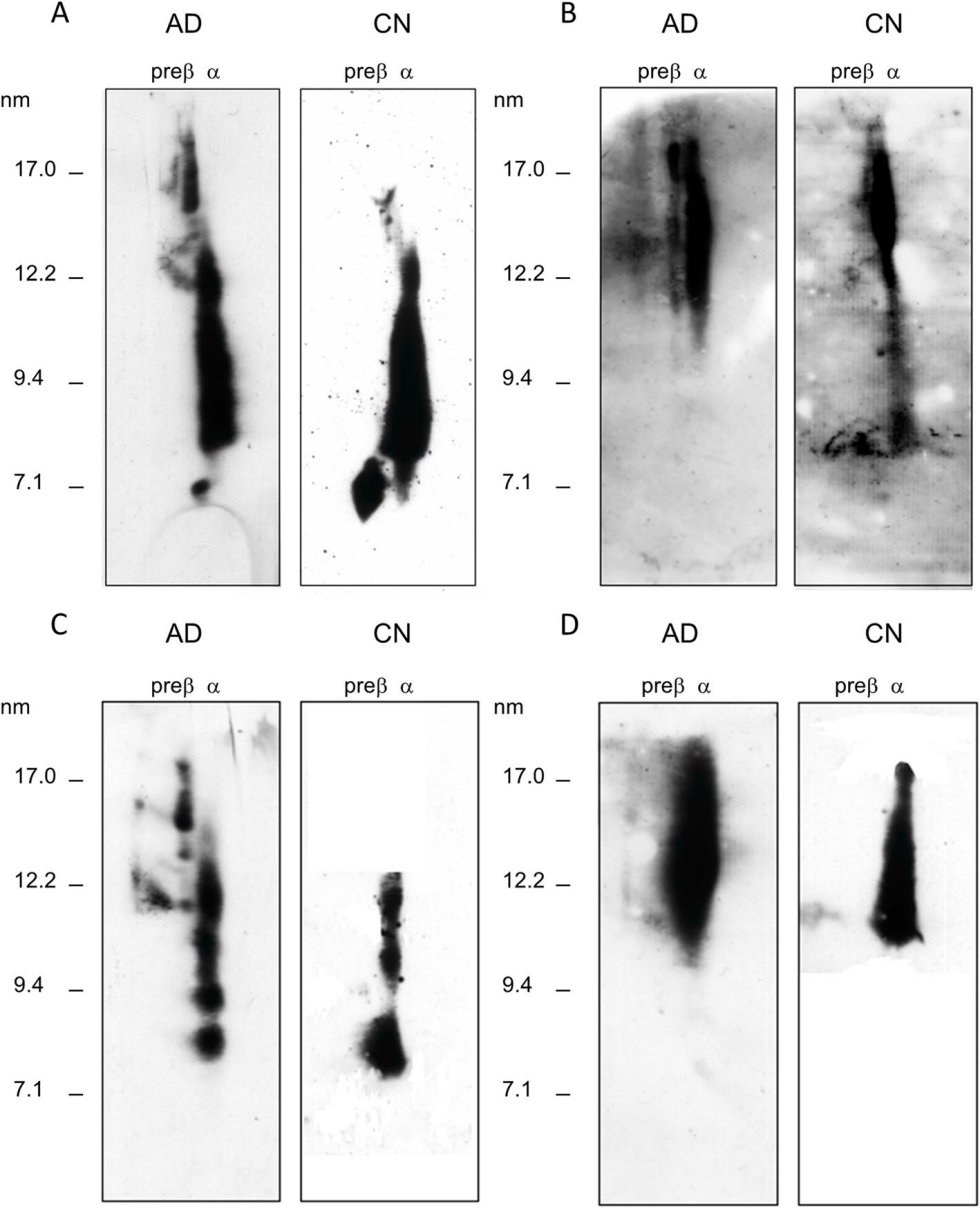
HDL subclass distribution in plasma and CSF of AD patients. Plasma (**A**, **B**) and CSF (**C**, **D**) HDL were separated by 2-D electrophoresis and detected by anti-apoA-I (**A** and **C**) or anti-apoE (**B** and **D**) antibodies. Images report representative HDL patterns of one AD patient (AD) and one control (CN). CSF was concentrated 10 folds before analysis

Plasma lipid and apolipoprotein levels were comparable between AD patients and controls, except for plasma unesterified cholesterol concentration, which was significantly lower in AD patients, thus leading to a strongly reduced unesterified/total cholesterol ratio (Table 2). Differently from previously reported data [1], plasma HDL-cholesterol, apoA-I, and apoA-II levels were in the normal range in AD patients. Plasma HDL subclass distribution in AD patients was comparable to that of controls (Fig. 1A, B), but the content of small discoidal preβ-HDL particles was reduced in AD patients (Table 1 and Fig. 1).

**Table 2:** Plasma lipid/lipoprotein profile of Alzheimer’s disease patients and control subjects

	AD (*n* = 70)	CN (*n* = 74)	*P*
Total cholesterol (mg/dL)	193.7 ± 44.3	198.4 ± 39.7	0.64
Unesterified cholesterol (mg/dL)	44.0 ± 15.7	56.0 ± 9.8	< 0.0001
Unesterified/total cholesterol	0.23 ± 0.08	0.28 ± 0.03	0.0002
Cholesteryl esters (mg/dL)	251.3 ± 70.1	244.9 ± 54.4	0.66
LDL cholesterol (mg/dL)	117.9 ± 38.6	106.9 ± 32.1	0.06
HDL cholesterol (mg/dL)	54.0 ± 16.1	57.1 ± 12.1	0.11
Non-HDL cholesterol (mg/dL)	139.7 ± 40.9	137.9 ± 36.3	0.78
Triglycerides (mg/dL)	105.0 (82; 129)	106.5 (75; 123)	0.95
Phospholipids (mg/dL)	209.2 ± 39.8	212.3 ± 42.1	0.94
Apolipoprotein A-I (mg/dL)	143.2 ± 27.8	138.8 ± 20.9	0.29
Apolipoprotein A-II (mg/dL)	28.6 ± 4.7	29.1 ± 7.3	0.83
Apolipoprotein E (mg/dL)	3.4 ± 1.1	2.9 ± 0.8	0.37
Apolipoprotein B (mg/dL)	96.7 ± 24	110.9 ± 31.4	0.03
LCAT activity (nmol/mL/h)	29.4 ± 12.4	36.4 ± 7.8	< 0.0001
CER (nmol/mL/h)	30.2 ± 12.9	35.9 ± 12.0	0.02
Preβ-HDL (% of total apoA-I)	9.95 ± 6.40	13.63 ± 4.12	0.03
Aqueous diffusion CEC (%)	6.7 ± 1.1	5.3 ± 1.0	< 0.0001
ABCG1 CEC (%)	4.8 ± 0.8	5.7 ± 1.5	0.0004
ABCA1 CEC (%)	3.7 ± 0.8	4.4 ± 1	0.001

*AD* Alzheimer’s disease, *CN* cognitive normal controls, *LCAT* lecithin:cholesterol acyltransferase, *CER* cholesterol esterification rate, *CEC*, cholesterol efflux capacity, *ABCG1* ATP-binding cassette G1, *ABCA1* ATP-binding cassette A1

Data are reported as mean ± SD or median (1^st^; 3^rd^ quartile). AD and controls were compared by the Wilcoxon rank-sum test

The evaluation of the plasma cholesterol esterification process revealed a partly compromised efficiency of the system in AD patients; LCAT activity and CER were reduced by 29% and 16%, respectively, in AD patients (Table 2). These results are in line with the strongly reduced plasma levels of unesterified cholesterol, the LCAT substrate.

Adjustment for statin use minimally affected the comparisons between plasma lipids in AD patients and controls, except for a shift to borderline significance for LDL-cholesterol (from *p* = 0.06 to *p* = 0.04) and an opposite shift for CER (from *p* = 0.02 to *p* = 0.12).

Interestingly, plasma unesterified cholesterol levels and the unesterified/total cholesterol ratio were significantly and positively associated with CSF Aβ_1-42_ content (*R* = 0.31, *p* = 0.010 and *R* = 0.29, *p* = 0.016, respectively) in the AD group (Supplemental Fig. 1).

Plasma HDL cholesterol efflux capacity was also evaluated in this cohort of subjects, as a metric of HDL function [30]. Aqueous diffusion cholesterol efflux capacity of plasma was higher in AD patients that in controls, but the two active CEC pathways, mediated by the transporters ABCA1 and ABCG1, were reduced in AD patients (Table 2), in agreement with the reduced content of discoidal pre-β HDL particles (Table 2), the major cholesterol acceptor via these pathways.

### CSF lipid/lipoprotein profile in AD patients

Lipids and apolipoproteins were measured in the CSF of all AD patients and in the CSF of a subgroup of cognitively normal controls (*n* = 21) and results are reported in Table 3. Interestingly, the CSF unesterified to total cholesterol ratio was increased in AD patients (Table 3); phospholipid CSF content was also increased (Table 3). As shown for plasma, CER was reduced in the CSF of AD patients (Table 3). When AD patients were divided in carriers or non-carriers of the *APOE* ε4 allele, CSF unesterified cholesterol was significantly lower in *APOE* ε4 carriers (0.14 ± 0.03 mg/dL vs. 0.17 ± 0.04 mg/dL, *p* = 0.03). ApoE content was also significantly increased in the CSF of AD patients compared to controls (Table 3), while no differences were observed in apoA-I CSF content between AD patients and controls (Table 3). In the AD group, apoA-I and apoE content in the CSF was significantly and positively correlated with CSF Aβ_1-42_ (R = 0.23, *p* = 0.05 and R = 0.47, *p* = 0.0001, respectively).

**Table 3:** Lipids/apolipoproteins in CSF of Alzheimer’s disease patients and control subjects

	AD (*n* = 70)	CN (*n* = 21)	*P*
Total cholesterol (mg/dL)	0.34 ± 0.13	0.40 ± 0.12	0.12
Unesterified cholesterol (mg/dL)	0.15 ± 0.04	0.15 ± 0.04	0.48
Unesterified/total cholesterol	0.49 ± 0.13	0.40 ± 0.12	0.07
Cholesteryl esters (mg/dL)	0.31 ± 0.18	0.42 ± 0.19	0.10
Phospholipids (mg/dL)	0.20 ± 0.08	0.14 ± 0.03	0.01
Apolipoprotein A-I (mg/dL)	0.91 ± 0.44	0.86 ± 0.28	0.99
Apolipoprotein E (mg/dL)	2.13 (1.5; 2.9)	1.35 (1.2; 1.8)	0.007
CER (nmol/mL/h)^a^	0.32 (0.15; 0.50)	2.27 (0.50–4.45)	0.05
CEC from astrocytes (%)	2.19 ± 0.60	2.84 ± 0.93	0.01

*AD* Alzheimer’s disease, *CN* cognitive normal controls, *CER* cholesterol esterification rate, *CEC* cholesterol efflux capacity

Data are reported as mean ± SD or median (1^st^; 3^rd^ quartile). AD and controls were compared by the Wilcoxon rank-sum test

^a^CER was measured in a subgroup of CSF samples (*n* = 10 for AD and *n* = 5 for CN)

CSF lipoprotein evaluation by 2-D electrophoresis revealed the predominance of apoE-containing particles but showed the presence of apoA-I-containing particles, with size and distribution very similar to that observed in plasma except for the absence of discoidal preβ-HDL (Fig. 1C, D).

Finally, we found that CSF CEC from astrocytes was significantly reduced in AD patients compared to controls (Table 3).

## Discussion

Brain contains a huge amount of cholesterol, 70% of which is present in myelin. The remaining 30% of brain cholesterol is distributed between glial cells and neurons, which in the mature state lose their ability to produce cholesterol and must rely for cholesterol supply on glial cells, mainly astrocytes [31]. Cholesterol transport in the brain is handled by lipoproteins very similar to circulating HDLs, mainly synthesized in situ but also deriving from the periphery [1]. Brain lipoproteins undergo a remodeling process like the one occurring in plasma, mediated by enzymes and lipid transfer proteins which are also expressed in the brain [1].

Cholesterol esterification is a fundamental step in cholesterol transport in plasma and indeed 70% of circulating cholesterol is esterified. Cholesterol esterification in humans is operated by three distinct enzymes: LCAT, which acts in plasma and other biological fluids, and sterol O-1 and sterol O-2 acyltransferases (SOAT1 and SOAT2), which are intracellular enzymes. LCAT is expressed mainly in the liver and is responsible for the formation of most of the plasma CEs, as shown by the almost absence of CEs in genetic LCAT deficiency [32]. SOAT1 is expressed in all cell types, while SOAT2 is expressed in hepatocytes and enterocytes, and in humans it has little contribution to circulating CEs [33]. Dysregulation of cholesterol metabolism in the brain has been associated with AD and other neurodegenerative disorders [1], but the mechanism(s) linking cholesterol metabolism and disease pathogenesis are not well understood.

Here we show for the first time that cholesterol esterification is altered in plasma and CSF of AD patients, and that these alterations strongly correlate with CSF AD biomarkers. LCAT is the enzyme responsible of almost all circulating CEs, thus abnormalities in plasma cholesterol esterification parameters (i.e., unesterified cholesterol levels and unesterified/total cholesterol ratio) likely derive from dysregulation of the LCAT system. Indeed, we have found reduced LCAT activity in the plasma of AD patients, which could be explained by a selective reduction of plasma unesterified cholesterol, the LCAT substrate, in the presence of normal concentrations of total cholesterol. Unesterified cholesterol is carried by all plasma lipoproteins, but mostly by small discoidal HDL particles, which are selectively reduced in AD patients’ plasma. Unesterified cholesterol in discoidal HDLs primarily derives from cell cholesterol efflux via the active ABCA1- and ABCG1-mediated pathways [34]. In the present cohort of AD patients, we have observed a reduced capacity of patients’ HDLs to promote transporter-mediated cell cholesterol efflux through these two pathways. The cholesterol esterification process in the brain is not well known, and the very low concentration of LCAT in the CSF makes it difficult to measure the process ex vivo. Using assays set for measurement in plasma appropriately modified, we first measured the CSF unesterified/total cholesterol ratio and the cholesterol esterification rate in CSF of cognitively normal controls and showed that CSF cholesterol esterification rate is much lower than in plasma, leading to a higher unesterified/total cholesterol ratio. The reduced CE formation in the CSF could be explained by the low LCAT concentration, and/or by the different substrate and activator. Indeed, small discoidal HDLs, the best LCAT substrate, are absent in the CSF, as shown by our HDL subclass analysis, and apoE, the most abundant apolipoprotein and likely LCAT activator in the CSF, is much less efficient than apoA-I in activating LCAT [35]. We next measured cholesterol esterification in the CSF of AD patients and found that CER was strongly reduced compared to controls, and the unesterified/total cholesterol was strongly increased in the CSF of AD patients, where around 50% of cholesterol is not esterified. As in the plasma, most of the CEs in the CSF should be LCAT-derived, thus suggesting a dysregulation of the LCAT system in AD CSF, which could be partly explained by the presence of unfunctional apoE in CSF of AD patients [36]. The lower content of CEs in AD CSF could also derive from the accumulation of CEs in the brain, and indeed excess CEs have been shown in brain vulnerable regions of AD patients [37]. The dysregulated esterification process may consequently halt the correct remodeling of HDL particles, with a negative impact on their cholesterol transport activity in the brain. In line with this hypothesis, we found that these particles were less efficient in promoting astrocyte cholesterol efflux, the first step of the HDL-promoted cholesterol transport towards neurons [27].

All together our results point to novel lipid-related factors implicated in AD, to be potentially used in the future as CSF or plasma biomarkers, in addition or as an alternative to those previously considered. Among these, total cholesterol and levels of HDL apolipoproteins, i.e., apoE, apo A-I, and apo J, have been already investigated [38, 39], leading to contradictory results on their association with AD [15, 40–43]. Similarly, oxysterols, in particular the neuronal-derived 24S-hydroxycholesterol, have been proposed as markers of neurodegeneration in AD, although with conflicting results [44–46]. Overall, these inconsistencies highlight the importance of identifying more reliable and powerful lipid biomarkers, that focus on peculiar and novel aspects of cholesterol metabolism, such as esterification.

## Conclusions

In conclusion, we found that cholesterol esterification is hampered in plasma and CSF of AD patients and that plasma cholesterol esterification biomarkers (unesterified cholesterol and unesterified/total cholesterol ratio) are significantly associated to disease biomarkers (i.e., CSF Aβ_1-42_). Such impairment is associated to defects in HDL functions, namely plasma and CSF cholesterol efflux capacity. Whether the dysregulation of cholesterol esterification is limited to AD or is a common trait in other neurodegenerative disorders remains to be defined. Taken together, the reported results suggest that LCAT-mediated cholesterol esterification could represent a potential target of novel therapeutic interventions in AD, although the causality between esterification abnormalities and related cognitive decline cannot be established from our data. A final caveat should be made, since only about a third of plasma samples were obtained from cognitively intact controls who were characterized by normal CSF biomarkers. In fact, albeit asymptomatic, some control subjects could express AD biomarkers many years before cognitive complaints may appear, possibly partially biasing our results.

### Supplementary Information

**Additional file 1: Supplementary Fig. 1.** Correlation plot between CSF-Aβ1-42 and plasma unesterified cholesterol (panel A) and between CSF-Aβ1-42 and unesterified/total cholesterol ratio (panel B) in AD patients. Confidence interval set at 95% (shadowed area) and reference line (dashed interval). Correlation coefficient is R = 0.31, *p* = 0.010 for A and R = 0.29, *p* = 0.016 for B.

## Data Availability

The datasets used and analyzed during the current study are available from the corresponding author on reasonable request.

## Abbreviations

ABCA1: ATP-binding cassette A1
ABCG1: ATP-binding cassette G1
Apo: Apolipoprotein
CEs: Cholesteryl esters
CEC: Cholesterol efflux capacity
CER: Cholesterol esterification rate
HDLs: High-density lipoproteins
LCAT: Lecithin:cholesterol acyltransferase
