# Supplementary material for: Plasma and cerebrospinal fluid cholesterol esterification is hampered in Alzheimer’s disease
Source: Alzheimers Res Ther. 2023 May 20;15:95. doi: 10.1186/s13195-023-01241-6 (PMC10199596; doi:10.1186/s13195-023-01241-6)
Supplement: Supplementary file 1 — Additional file 1: Supplementary Fig. 1. Correlation plot between CSF-Aβ1-42 and plasma unesterified cholesterol (panel A) and between CSF-Aβ1-42 and unesterified/total cholesterol ratio (panel B) in AD patients. Confidence interval set at 95% (shadowed area) and reference line (dashed interval). Correlation coefficient is R = 0.31, p = 0.010 for A and R = 0.29, p = 0.016 for B. [file 13195_2023_1241_MOESM1_ESM.pdf]

Plasma and cerebrospinal fluid cholesterol esterification is hampered in Alzheimer's Disease

Marta Turri[1], Elisa Conti[2], Chiara Pavanello[1], Francesco Gastoldi[1], Marcella Palumbo[3], Franco Bernini[3], Vittoria Aprea[2], Francesca Re[4], Alberto Barbiroli[5], Davide Emide[5], Daniela Galimberti[6,7], Lucio Tremolizzo[2*], Francesca Zimetti[3*], Laura Calabresi[1*]; AGAINST-AD Group

[1]Centro E. Grossi Paoletti, Dipartimento di Scienze Farmacologiche e Biomolecolari, Università degli Studi di Milano, Milano, Italy; [2]Neurology Unit, IRCCS "San Gerardo dei Tintori", Monza and University of Milano-Bicocca, Italy; [3]Department of Food and Drug, University of Parma, Parma, Italy; [4]School of Medicine and Surgery, University of Milano-Bicocca, Italy; [5]Dipartimento di Scienze per gli Alimenti, la Nutrizione e l'Ambiente, Università degli Studi di Milano, Italy; [6]Department of Biomedical, Surgical and Dental Sciences, University of Milan, Italy; [7]Fondazione IRCCS Ca' Granda, Ospedale Policlinico, Milan, Italy

*These authors equally contributed

Corresponding author:
Laura Calabresi, PhD
Center Grossi Paoletti, Department of Pharmacological and Biomolecular Sciences, University of Milano
Via Balzaretti 9, 20133 Milano, Italy
Phone: +390250319906
Fax: +390250319900
E-mail: laura.calabresi@unimi.it

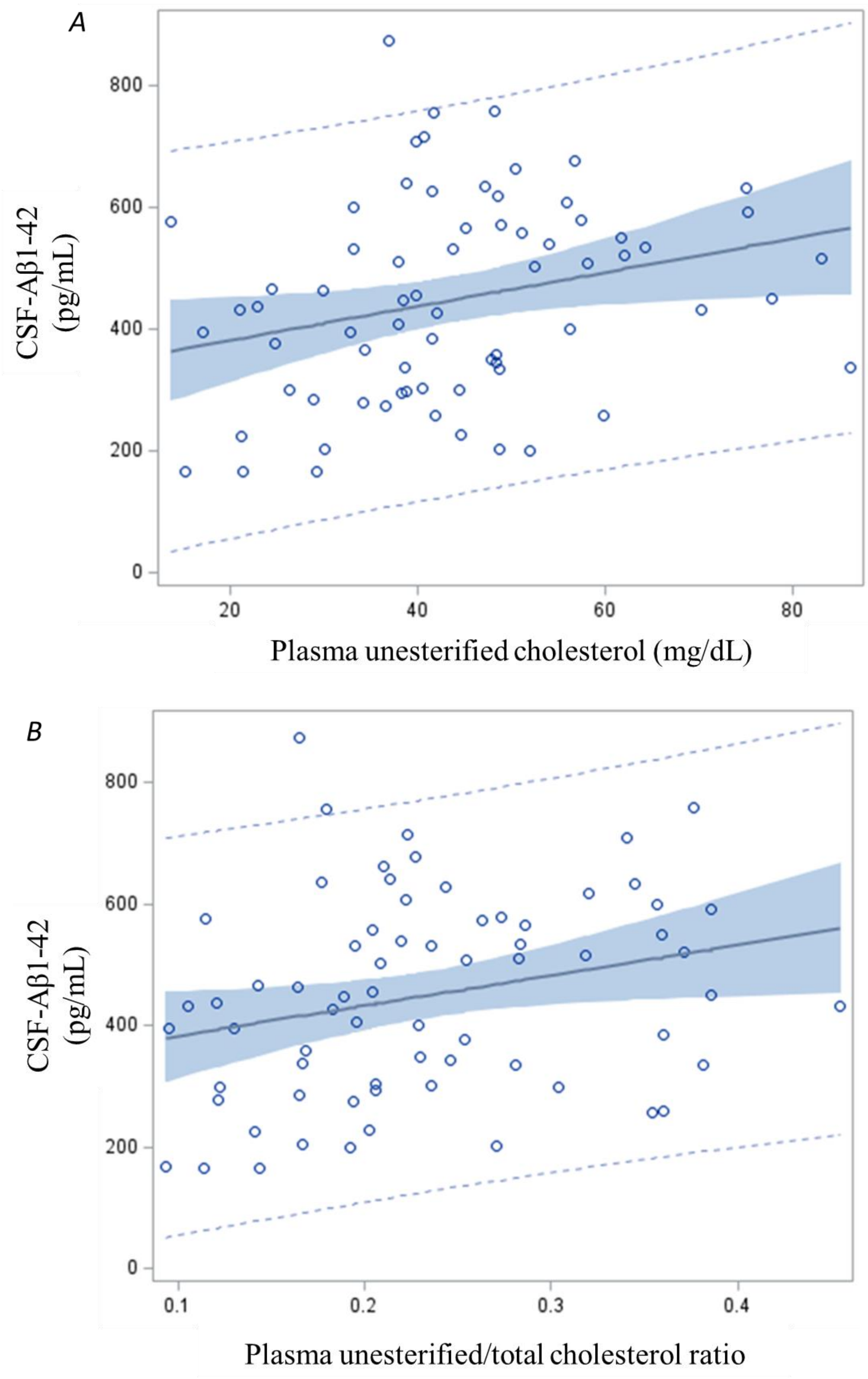

Supplementary Fig. 1. Correlation plot between CSF-A$\beta_{1\text{-}42}$ and plasma unesterified cholesterol (*panel A*) and between CSF-A$\beta_{1\text{-}42}$ and unesterified/total cholesterol ratio (*panel B*) in AD patients. Confidence interval set at 95% (shadowed area) and reference line (dashed interval). Correlation coefficient is R=0.31, *p*=0.010 for *A* and R=0.29, *p*=0.016 for *B*.
